# Diversity of the protease-producing bacteria and their extracellular protease in the coastal mudflat of Jiaozhou Bay, China: in response to clam naturally growing and aquaculture

**DOI:** 10.3389/fmicb.2023.1164937

**Published:** 2023-05-19

**Authors:** Zhiyun Liu, Guangchao Liu, Xuzhen Guo, Yang Li, Na Ji, Xingfeng Xu, Qingjie Sun, Jie Yang

**Affiliations:** ^1^College of Food Science and Engineering, Qingdao Agricultural University, Qingdao, China; ^2^College of Life Science, Qingdao Agricultural University, Qingdao, China; ^3^Qingdao Special Food Research Institute, Qingdao, China

**Keywords:** bacterial community, protease-producing bacteria, diversity, clam naturally growing area, clam aquaculture area, coastal mudflats

## Abstract

The booming mudflat aquaculture poses an accumulation of organic matter and a certain environmental threat. Protease-producing bacteria are key players in regulating the nitrogen content in ecosystems. However, knowledge of the diversity of protease-producing bacteria in coastal mudflats is limited. This study investigated the bacterial diversity in the coastal mudflat, especially protease-producing bacteria and their extracellular proteases, by using culture-independent methods and culture-dependent methods. The clam aquaculture area exhibited a higher concentration of carbon, nitrogen, and phosphorus when compared with the non-clam area, and a lower richness and diversity of bacterial community when compared with the clam naturally growing area. The major classes in the coastal mud samples were Bacteroidia, Gammaproteobacteria, and Alphaproteobacteria. *The Bacillus*-like bacterial community was the dominant cultivated protease-producing group, accounting for 52.94% in the non-clam area, 30.77% in the clam naturally growing area, and 50% in the clam aquaculture area, respectively. Additionally, serine protease and metalloprotease were the principal extracellular protease of the isolated coastal bacteria. These findings shed light on the understanding of the microbes involved in organic nitrogen degradation in coastal mudflats and lays a foundation for the development of novel protease-producing bacterial agents for coastal mudflat purification.

## Introduction

The coastal mudflat is a complex and dynamic ecological system, which is deeply influenced by the geological, physicochemical, and biological factors of marine and terrestrial land. Previous studies have reported that the spatiotemporal dynamics of bacterial abundance, diversity, and activity in tidal flat sediments were significantly affected by the biogeochemical heterogeneity of salinity, pH, carbon, nitrogen, sulfur, and phosphorus (Taylor et al., 2014; Soares et al., 2018; Zhang G. et al., 2021; Niu et al., 2022; Zhang et al., 2022). The seasonality and sediment depth also affected the bacterial communities in the mudflat (Böer et al., 2009; Gobet et al., 2012). Moreover, bacteria were found to be deeply involved in carbon fixation and carbon-containing compound degradation, as well as nitrogen cycling processes including nitrogen fixation, ammonium oxidation, and nitrite and nitrous oxide reduction in the coastal mudflats (Hou et al., 2013; Zhou et al., 2018; Li et al., 2021; Li Q. et al., 2022).

Coastal mudflats are of great significance in regulating regional climate and sustaining ecological balance. In recent years, mudflat planting, mudflat aquaculture, and eco-tourism are flourishing in coastal areas, which have promoted economic growth, but also posed serious environmental threats (Long et al., 2016). With the expansion of human activities, the contents of organic carbon and nitrogen in coastal mudflats were significantly increased, resulting in a large amount of organic matter entering the sea and the atmosphere (Hu et al., 2019; Li T. et al., 2022). The nitrogen cycle is a main component of global biochemical cycles, and the nitrogen budget plays an important role in keeping ecological balance (Zhang X. et al., 2020; Hutchins and Capone, 2022). Current studies of bacterial functions in the nitrogen cycle mainly focused on nitrogen fixation, anaerobic ammonia oxidation, nitrification, and denitrification (Zhou et al., 2018; Li et al., 2021; Li Q. et al., 2022).

Protease is an important participant in the degradation of organic nitrogen, and protease-producing bacteria have been reported as the main microflora that regulate the nitrogen content in ecosystems (Zhou et al., 2013; Zhang et al., 2015; Tornkvist et al., 2019; Zhang Y. Z. et al., 2020). Related studies based on culture-dependent methods have been conducted in a tropical aquaculture environment (Wei et al., 2021), in the coastal sediments and soils of Antarctica (Zhou et al., 2013; Liu et al., 2021), the sediments of the Bohai Sea, Yellow Sea, and South China Sea (Zhou et al., 2009; Zhang et al., 2019), and the sediments of Jiaozhou Bay (Zhang et al., 2015), showing the diversity of protease-producing bacteria and their extracellular protease in various environments. In addition, a variety of microorganisms with enzyme-producing capacities have been isolated from coastal mudflats (Das, 2012; Suthindhiran et al., 2013; Gaonkar and Furtado, 2020). To the best of our knowledge, reports on the diversity of protease-producing bacteria in coastal mudflats are relatively rare. We speculated that an abundant and diverse protease-producing bacteria community existed in the coastal mudflats, especially in mudflat aquaculture with a high content of organic nitrogen. Therefore, it is necessary to study the diversity of protease-producing bacteria in coastal mudflats that are heavily influenced by anthropogenic activity.

Jiaozhou Bay is a representative semi-enclosed bay of China's Yellow Sea. Under the influence of dense and long-time human activities, the ecological factors of Jiaozhou Bay have changed significantly (Zhang L. et al., 2021; Lin et al., 2022). Eutrophication and high organic nitrogen content occurred in the coastal sediments of the bay due to terrestrial inputs (Liu et al., 2010; Li H. et al., 2019; Zhang L. et al., 2021), all of which make it an ideal area for studying the organic nitrogen biodegradation in the coastal mudflats. In this study, mud samples were collected from three stations in the coastal mudflat of Jiaozhou Bay, including the non-clam area, the clam naturally growing area, and the clam aquaculture area. The variation of bacterial taxonomy composition of different loci was investigated by culture-independent methods. In addition, protease-producing bacteria were isolated from these samples by culture-dependent methods, and the diversity of both the bacteria and the extracellular proteases as well as their distribution in different stations were further analyzed. Our research provides experimental evidence to elucidate the ecological role of microorganisms in organic nitrogen degradation by decomposing protein in coastal mudflats and lays a foundation for the development of measures to protect the coastal mudflat.

## Materials and methods

### Sampling and geochemical characteristics detection

Mud samples were collected from the coastal mudflat of Jiaozhou Bay, including three stations: the non-clam area (sample D1-3, hereafter collectively referred to as sample D), the clam naturally growing area (sample F1-3, hereafter collectively referred to as sample F), and the clam aquaculture area (sample H1-3, hereafter collectively referred to as sample H) on 18 March 2021. The geographic location of sampling sites is shown in Supplementary Figures S1–S4. The surface mud of each station was removed, and then, a deeper mud sample (5–10 cm) was collected aseptically using stainless-steel sterile shovels. Three replicates of all samples were taken from each station. The collected mud samples were stored in airtight sterile plastic bags at 4°C for subsequent geochemical characteristics detection and microbiological analysis. The salinity of the mud samples was measured using a soil salt meter PNT3000 (STEPS, Germany). The pH, total carbon content (TC), total nitrogen content (TN), and total phosphorus content (TP) of each sample were measured according to NY/T 1121.2-2006, NY/T1121.6-2006, NY/T1121.24-2012, and NY/T88-1988, respectively. The paired sample *t*-test was applied to evaluate the statistical significance between different variables of this study. The level of significance was set to a *p*-value of < 0.05.

### DNA extraction, Illumina sequencing, and analysis

The total genomic DNA of the mud samples was extracted with OMEGA-soil DNA Kit (Omega Bio-Tek, USA). The V3–V4 regions of bacterial 16S rRNA gene were amplified using TransStart FastPfu Polymerase (TransGen Biotech, China), with Barcoded sequencing primers 338F (5′-ACTCCTACGGGAGGCAGCA-3′) and 806R (5′-GGACTACHVGGGTWTCTAAT-3′). The generated PCR mixtures were then sequenced on an Illumina Miseq PE300 sequencing platform (Shanghai Majorbio, China). The obtained large short-read libraries were merged and trimmed, and the standard quality control was performed with Usearch 8.0.161 by the sequencing company. The remaining reads of the three samples were then pooled, dereplicated, and finally assigned to each OTU using a 97% identity. OTU clustering and taxonomy assignment were processed by mapping the reads of each sample to representative OTU sequences using a Perl script, followed by comparing with a 16S rRNA gene database (RDP Release 11.1 http://rdp.cme.msu.edu/) at an 80% confidence threshold. The data were analyzed on the online Majorbio Cloud Platform (www.majorbio.com) developed by Shanghai Majorbio Bio-pharm Technology Co. Ltd. (Ren et al., 2022). Statistical analysis of alpha diversity was conducted via *t*-test at a significance level of 5% (*p* < 0.05).

### Screening of protease-producing bacteria

Synthetic sea salt was purchased from Qingdao Sea-Salt Aquarium Technology Co. Ltd. and then dissolved at a concentration of 3% to make artificial seawater. The screening solid medium was prepared by adding 1% casein, 2% gelatin, 0.2% yeast extract, and 1.5% agar into artificial seawater with a final pH of 8.0. Protease-producing bacteria were screened using the dilution-plate method according to previous studies, with minor modifications (Zhang et al., 2015; Liu et al., 2021). In brief, approximately 1 g of mud sample was diluted serially 10 times to 10^−6^ using artificial seawater. Aliquots of 100 μl of each diluted sample were spread on the screening plates for subsequent incubation at 20°C until visible hydrolytic zones were formed. Colonies of different morphology with hydrolytic zone were further repeatedly streaked on the same medium at least three times to obtain a pure strain.

### 16S rRNA gene amplification, sequencing, and phylogenetic analysis

The *16S rRNA* gene of culturable bacteria was amplified by colony PCR technique using a Colony PCR kit (Mei5 Biotechnology, China) with the universal primers 27F (5′-AGAGTTTGATCCTGGCTCAG-3′) and 1492R (5′-GGTTACCTTGTTACGACTTC-3′), and then, the PCR products were sequenced by Shanghai Tsingke Biotechnology Co. Ltd., China. Isolates with at least two different nucleotides in their 16S rRNA gene sequences were identified as different strains. Phylogenetic trees were constructed using MEGA 11 with the neighbor-joining method. The GenBank accession numbers of 16S rRNA gene sequences in this study were OQ625902, OQ625903, OQ625905–OQ625912, OQ625916, OQ625917, OQ625921, OQ625938, OQ625939, OQ625941, OQ625993–OQ625996, OQ626000–OQ626019, OQ626205-OQ626214, OQ626220, OQ626221, OQ626228, OQ629890, OQ629902, OQ629992–OQ629994, OQ642131, OQ651245, and OQ690495. The strains' corresponding accession numbers are shown in Supplementary Table 1.

### Protein substrate specificity test

The protease hydrolysis ability of the pure strains toward different substrates, including milk powder, casein, and gelatin, was detected using the method of Zhou et al. (2009), with minor modifications. Protein substrate solid media were prepared by mixing each protein substrate (1.0% milk powder, 0.5% casein, or 0.5% gelatin) with 0.2% yeast extract, 1.5% agar, and artificial seawater and adjusting the final pH to 8.0. The bacterial strains were streaked on the substrate plates and then incubated at 20°C for 4 days. The specific value of the diameter of the formed hydrolytic zone divided by the diameter of the colony, and the H/C ratio was measured and calculated.

### Protease inhibitor assay

The bacterial strain was cultured in a liquid screening medium at 20°C and 200 rpm for 4 days. The supernatant of the bacterial culture was collected after centrifugation at 12,000 rpm at 4°C for 10 min and then used for subsequent protease inhibitor assay. The inhibitors contained phenylmethylsulfonyl fluoride (PMSF, Sigma) at a concentration of 1.0 mM, 1,10-phenanthroline (OP, Sigma) at a concentration of 1.0 mM, E64 (Merck) at a concentration of 0.1 mM, and pepstatin A (PA, Merck) at a concentration of 0.1 mM. The supernatant was properly diluted with 50 mM Tris-HCl (pH 8.0), followed by incubating with each inhibitor at 4°C for 60 min, and then, the residue protease activity was measured by the digestion of casein as formerly described (Chen et al., 2003). In brief, 100 μl of diluted solution and 100 μl of 2% casein in 50 mM Tris-HCl (pH 8.0) were mixed and incubated at 25°C for 20 min. To stop the reaction, 200 μl of 0.4 M TCA was added to the reaction mixture. After centrifugation at 12,000 rpm and 4°C for 5 min, the supernatant was collected and every 100 μl of supernatant was incubated with 500 μl of 0.4 M Na_2_CO_3_ and 100 μl of Folin–Ciocalteu's phenol reagent at 40°C for 10 min, and then, the absorbance was measured at 660 nm. One unit of protease activity was defined as the amount of enzyme that released 1 μg of tyrosine per milliliter of reaction mixture per minute. A sample without the addition of an inhibitor was set as the negative control. The difference between the relative residue activity of each sample and the negative control was taken as the inhibition ratio (%).

## Results

### Geochemical characteristics of the mud samples

The mud samples were collected from three stations in a coastal mudflat of Jiaozhou Bay, including the non-clam area (sample D), the clam naturally growing area (sample F), and the clam aquaculture area (sample H). The results of the geochemical analysis are shown in Table 1. All mud samples exhibited slightly alkaline pH (8.21–8.74). The salinity ranged from 4.75 to 6.25 g/kg, with the highest observed in sample H and the lowest in sample D. The contents of TC, TN, and TP in the samples ranged from 9.64–22.42 g/kg (TC), 0.52–0.79 g/kg (TN), and 0.31–0.62 g/kg (TP), respectively. The highest values were found in sample H (TC and TP) and sample F (TN), while the lowest were found in sample D (TC, TN, and TP). The carbon content, nitrogen content, and phosphorus content in the clam naturally growing area and the clam aquaculture area were significantly higher than the corresponding values in the non-clam area, indicating that the artificial culture and growth of clams may be relevant to eutrophication in a tidal area.

**Table 1:** Characteristics of the mud sampling stations in the coastal mudflat of Jiaozhou Bay^1^.

**Station**	**Sample**	**Location**	**Temperature**	**pH**	**Salinity**	**TC**	**TN**	**TP**
		**(E, N)**	**(**°**C)**		**(g/kg)**	**(g/kg)**	**(g/kg)**	**(g/kg)**
Non-clam area	D	120°15'60”,	15.8	8.51 ± 0.11^a^	4.75 ± 0.18^a^	9.64 ± 0.04^a^	0.52 ± 0.01^a^	0.31 ± 0.02^a^
		36°10'55”						
Clam naturally growing area	F	120°16'1”,	15.8	8.74 ± 0.08^b^	5.85 ± 0.20^c^	16.39 ± 0.53^c^	0.79 ± 0.11^b^	0.42 ± 0.03^b^
		36°10'56”						
Clam aquaculture area	H	120°16'22”,	15.8	8.21 ± 0.15^b^	6.25 ± 0.31^c^	22.4 ± 0.08^c^	0.76 ± 0.04^c^	0.62 ± 0.10^b^
		36°11'6”						

^1^*T*-test was applied to evaluate the statistical differences between the studied variables compared to their corresponding variable of the non-clam area. Different letters in the same column are significantly different at a *p*-value of < 0.05 (ab) and at a *p*-value of < 0.01 (ac) between the non-clam area and the other stations.

### Bacterial taxonomy composition analysis

The microbial community of the mud samples was investigated by using culture-independent methods based on Illumina high-throughput sequencing. A total of 113,717 effective sequence reads were obtained, and the sequencing coverage rate exceeded 97%. A total of 5,135 OTUs were assigned, which were arranged from high to low as sample F (2,090), sample D (1,574), and sample H (1,471). The alpha diversity indexes are shown in Table 2. The estimators Ace and Chao reveal the richness of the bacterial community. Sample F showed higher Ace and Chao values than samples D and H, indicating higher bacterial richness in sample F. The estimators Shannon and Simpson express the diversity of the microbial community, with a positive correlation between Shannon and a negative correlation between Simpson. Sample F, again, exhibited the highest Shannon value and the lowest Simpson value, implying that the microbial diversity of Sample F was the highest of all the samples analyzed. The richness and diversity of the bacterial community in the clam naturally growing area were significantly higher than that of the clam aquaculture area and the non-clam area. However, the results of statistical analysis indicated that there are no statistically significant differences in microbial richness and diversity between the clam aquaculture area and the non-clam area.

**Table 2:** Alpha diversity estimators of the bacterial community in the mud samples^1^.

**Station**	**Sample**	**Coverage**	**OTUs**	**Bacterial richness index**		**Bacterial diversity index**	
		**(%)**		**Ace**	**Chao**	**Shannon**	**Simpsonx 10** ^−3^
Clam naturally growing area	F	97.74	2,055 ± 50^a^	2,509 ± 37^a^	2,469 ± 42^a^	6.28 ± 0.005^a^	5.72 ± 0.32^a^
Clam aquaculture area	H	97.95	1,493 ± 31^c^	2,087 ± 73^b^	2,005 ± 61^b^	5.47 ± 0.25^b^	19.15 ± 0.30^b^
Non-clam area	D	97.97	1,545 ± 41^c^	2,018 ± 74^b^	1,991 ± 104^b^	5.73 ± 0.01^c^	8.68 ± 0.36^b^

^1^Statistical analysis was made by *t*-test. Different lower cases in the same column represent significance at a *p-*value of < 0.05 (ab) and at a *p-*value of < 0.01 (ac) between the clam naturally growing area and the other studied stations.

**Figure 1:**
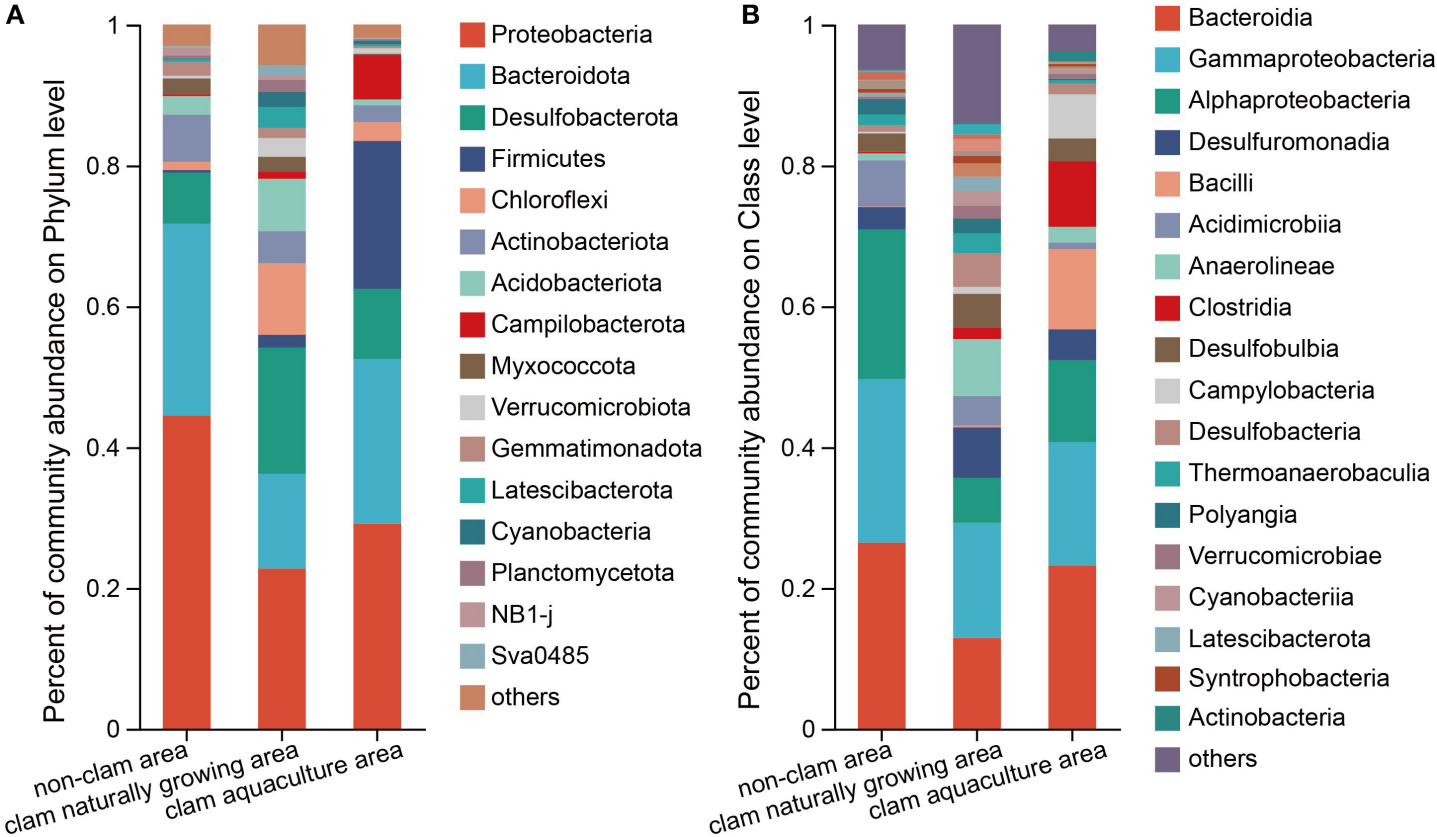
Composition of the bacterial community of the mud samples on phylum level **(A)** and class level **(B)**. The mud samples were taken from the non-clam area (sample D), the clam naturally growing area (sample F), and the clam aquaculture area (sample H). The microbial community was investigated by culture-independent methods. The column stands for different mud samples, and the row represents the relative percentage of each bacterial type, which is depicted by different colors.

In total, 52 phyla, 136 classes, 304 orders, 468 families, and 833 genera were identified in the three mud samples. Figure 1 shows the top phyla and classes of the mud samples. It seems that the bacterial diversity of sample F was the highest and that of H was the lowest (Figure 1), which was consistent with the above alpha diversity estimators. The phylum with more than 5% was considered the dominant phylum. Proteobacteria (44.47%) and Bacteroidota (27.26%) were definitively dominant in sample D, with a sum of 71.73% (Figure 1A), while in sample F, the major phyla were Proteobacteria (22.76%), Desulfobacterota (17.92%), Bacteroidota (13.44%), Chloroflexi (10.14%), and Acidobacteriota (7.45%), with a sum of 71.71% (Figure 1A). The dominant phyla constituted 89.76% of the bacterial community in sample H, which were Proteobacteria (29.13%), Bacteroidota (23.40%), Firmicutes (21.03%), Desulfobacterota (9.93%), and Campilobacterota (6.27%) (Figure 1A). On class level, Bacteroidia, Gammaproteobacteria, and Alphaproteobacteria were dominant in the mud samples, contributing to a sum of 70.92, 36.40, and 52.35% of the bacterial community in samples D, F, and H, respectively. It is noted that *Bacilli* with an abundance of 11.38% and *Clostridia* with an abundance of 9.34% were two major classes in sample H but were barely found in sample D and sample F (Figure 1B). On the genus level, *Woeseia* and *Loktanella* were the major bacterial genera in the non-clam area, *Woeseia* was dominant in the clam naturally growing area, and *Trichococcus, Lutibacter*, and *Psychrobacter* were dominant in the clam aquaculture area (Supplementary Figure 5).

### Diversity of cultivable protease-producing bacteria

Protease is a key factor involved in nitrogen recycling (Zaman et al., 1999; Kamimura and Hayano, 2000). To further study the microbe-associated organic nitrogen degradation in coastal mudflats, we examined the cultivable protease-producing bacterial communities in mud samples by using culture-dependent methods. Moreover, culture-dependent methods have been commonly used for the discovery of novel species and bacterial products, which may further develop as protease-producing microbial agents for depollution of the environment. In general, the cultivability of the microbial community in the coastal area ranges from 0.1 to 0.01% (Al-Mailem et al., 2014; Rajeev et al., 2019). A number of colonies appeared on the screening plates of the 10^−1^-10^−4^ diluted mud samples after cultivation. The bacterial abundance reached 10^4^ cells/g mud in the non-clam area (sample D) and the clam aquaculture area (sample H) and 10^5^ cells/g mud in the clam naturally growing area (sample F). Clear hydrolytic zones were found around approximately 60% of the colonies on the screening plates. These results indicated that a large quantity of protease-producing bacteria inhabited the studied coastal muds.

**Figure 2:**
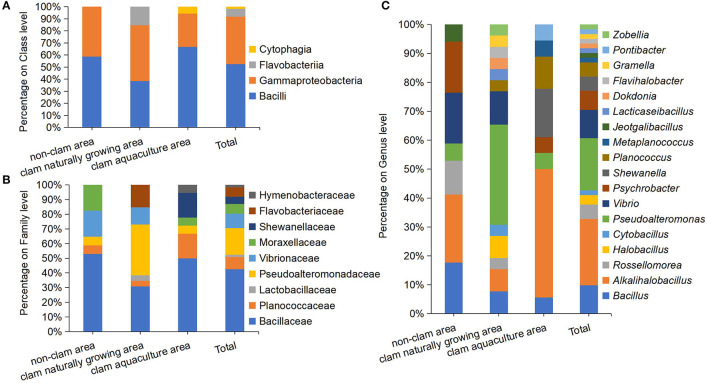
Diversity and distribution of culturable protease-producing bacteria of mud samples on class level **(A)**, family level **(B)**, and genus level **(C)**. The mud samples were taken from the non-clam area (sample D), the clam naturally growing area (sample F), and the clam aquaculture area (sample H), and the microbial community was investigated by culture-dependent methods. Each color represents the percentage of the taxon in the total isolates.

The pure strain of the above colonies was obtained by repeated streaking cultivation. The nearly full-length *16S rRNA* genes of the isolates were amplified and sequenced, and isolates with two or more different nucleotides in their *16S rRNA* gene were considered as different strains. Finally, a total of 61 different protein hydrolyzing bacterial strains were isolated; 17 strains from sample D, 26 strains from sample F, and 18 strains from sample H (Supplementary Table 2). The 61 strains were classified into three phyla, four classes, nine families, and 18 genera (Figure 2). On the phylum and class level of the culturable fraction, the phyla Firmicutes and Proteobacteria and the classes Bacilli and Gammaproteobacteria were the largest groups in the three studied areas (Figure 2A). On the family level of the culturable fraction, Bacillaceae was the major family in all the studied areas and the dominance of Pseudoalteromonadaceae exists only in the clam naturally growing area (Figure 2B). The family Bacillaceae contains *Bacillus*-like species, including the five genera *Bacillus, Alkalihalobacillus, Rossellomorea, Halobacillus*, and *Cytobacillus*, with an abundance of 52.94% in sample D, 30.77% in sample F, and 50% in sample H, implying a dominance of *Bacillus*-like bacteria in the analyzed culturable fraction from coastal mudflats (Figures 2B, C). *Pseudoalteromonas* dominated in sample F (34.62%), meanwhile only a minor genus dominated in sample D (5.88%) and sample H (5.6%) (Figure 2C). In addition, the isolated protease-producing bacteria of sample F affiliated with 13 genera were apparently more diverse than that of sample D with seven genera and sample H with eight genera (Figure 2C), which was consistent with the findings of bacterial diversity by the above culture-independent methods.

**Figure 3:**
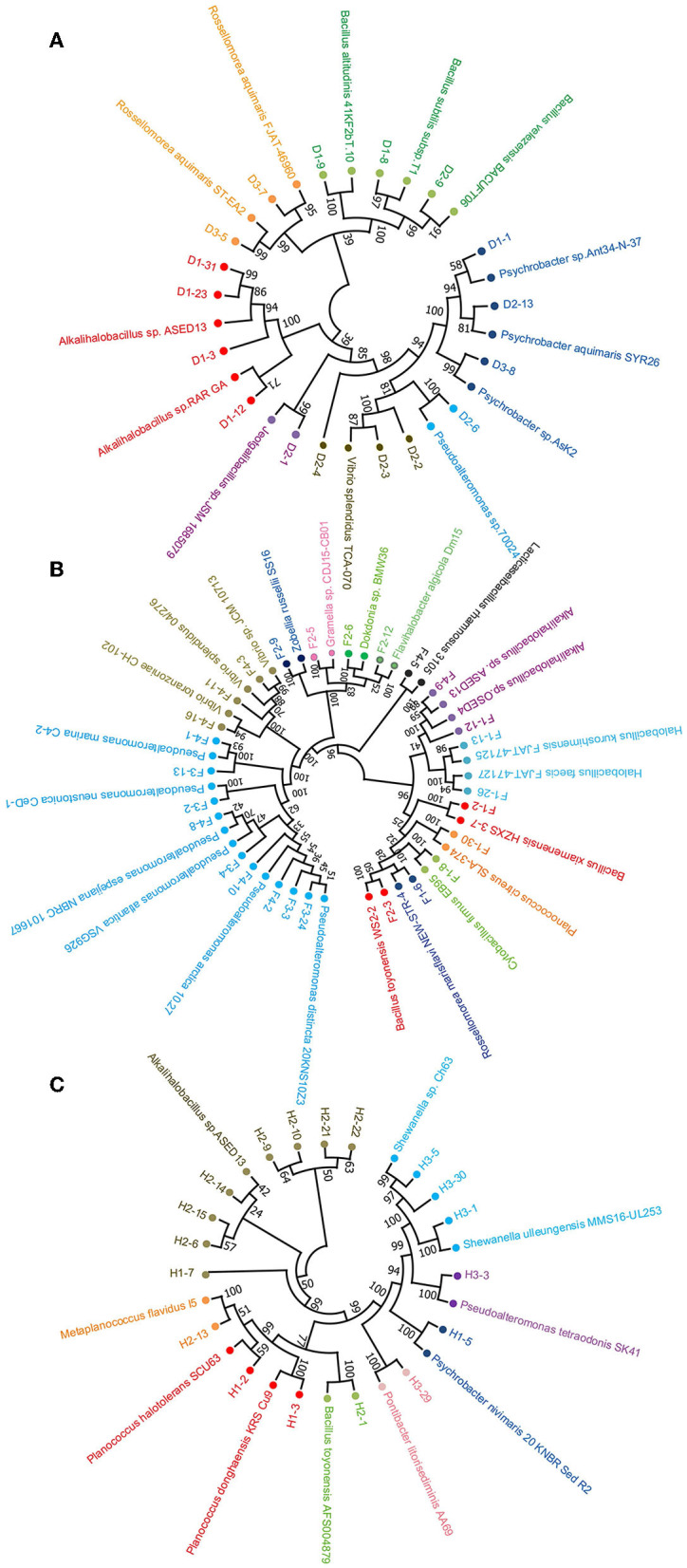
Neighbor-joining phylogenetic tree based on 16S rRNA sequencing of protease-producing bacteria isolated from three stations of coastal mudflat in Jiaozhou Bay using MEGA 11. The stations were the non-clam area **(A)**, the clam naturally growing area **(B)**, and the clam aquaculture area **(C)**. Strains affiliated with different genera are shown in different colors.

In addition, neighbor-joining phylogenetic trees of the protease-producing strains with different genera based on *16S rRNA* gene sequences were constructed (Figure 3). The relationship between the strains isolated from the non-clam area, the clam naturally growing area, and the clam aquaculture area is shown in Figures 3A–C, respectively. *Alkalihalobacillus* strain H1-7 illustrated a distant relationship with other recognized species and may represent a new taxonomic unit worthy of further research and investigation.

### Diversity analysis of bacterial extracellular proteases

Furthermore, the diversity of the bacterial extracellular protease was investigated by protein substrate specificity testing and protease inhibitor assay. Among the total 61 isolated strains, the extracellular proteases from 59, 55, and 55 strains could hydrolyze milk powder, casein, and gelatin, respectively, forming clear hydrolytic zones (Supplementary Table 2). The difference in the H/C ratios of the bacterial strains reflected their variation in substrate specificity. There was a certain variety in the activity of extracellular protease toward different protein substrates. The extracellular proteases secreted from *Alkalihalobacillus* strains F4-9 and H1-7, *Bacillus* strains D1-8, F1-2, and F2-3, *Halobacillus* strain F1-13, *Jeotgalibacillus* strain D2-1, and *Psychrobacter* strains H1-5 had high hydrolytic activity toward milk powder, with H/C ratios higher than five. The proteases from the *Alkalihalobacillus* strain D1-12, *Bacillus* strains D1-8 and F1-2, and *Planococcus* strain H1-3 showed high hydrolytic activity toward casein. The proteases from the *Bacillus* strain D2-9, *Halobacillus* strain F1-13, and *Psychrobacter* strains D2-13, D1-1, and H1-5 exhibited high hydrolytic activity toward gelatin.

In addition, the diversity of bacterial extracellular proteases was further investigated by detecting the inhibition ratios of PMSF (serine protease inhibitor), OP (metalloprotease inhibitor), Pepstatin A (aspartic protease inhibitor), and E64 (cysteine protease inhibitor). The 61 strains were cultivated in the liquid screening medium, and only 20 strains affiliated with the genera *Alkalihalobacillus, Bacillus, Planococcus, Rossellomorea, Halobacillus, Jeotgalibacillus, Psychrobacter, Pseudoalteromonas*, and *Shewanella* were able to produce enough extracellular proteases for protease inhibitor assay (Table 3). The extracellular protease activities of all 20 strains were inhibited by PMSF at the degree of 8.77–68.97%, indicating that all the coastal mudflat strains produced serine proteases. OP inhibited the protease activities of 16 strains by more than 10%, indicating that a majority of the isolated strains produced extracellular metalloproteases. In particular, an inhibition ratio as high as 84.97% was observed in the *Alkalihalobacillus* strain F1-12, suggesting that the *Alkalihalobacillus* strain F1-12 mainly produced extracellular metalloproteases. In contrast, Pepstatin A inhibited extracellular protease activity by 20% or less, indicating that these strains secrete very little aspartic or cysteine proteases. Moreover, E64 exhibited little inhibitory effect on the extracellular protease activities, with the exception of the *Alkalihalobacillus* strain F1-12, *Bacillus* strains D1-8 and D1-9, and *Halobacillus* strain F1-13, demonstrating that only a minority of the strains produced extracellular cysteine protease.

**Table 3:** Inhibition ratios of inhibitors on bacterial extracellular proteases.

**Phyla**	**Genera**	**Strains**	**Inhibition ratio (%)** ^**a**^			
			**PMSF (1.0 mM)**	**OP (1.0 mM)**	**Pepstatin A (0.1 mM)**	**E64 (0.1 mM)**
Firmicutes	Alkalihalobacillus	D1-3	28.83	52.28	11.66	6.1
		F1-12	36.58	84.97	10.87	25.59
		H1-7	24.31	29.13	13.72	4.93
		H2-9	8.77	28.02	—	—
	*Bacillus*	D1-8	38.56	56.64	2.59	21.82
		D1-9	53.12	19.51	18.94	22.03
		D2-9	48.08	4.24	—	—
		F2-3	18.01	57.52	20.06	11.66
	*Planococcus*	F1-30	37.17	41.78	4.53	1.67
		H1-3	53.21	18.68	—	—
		H1-2	61.22	33.83	—	—
	*Metaplanococcus*	H2-13	10.26	28.16	—	—
	*Rossellomorea*	D3-7	21.66	10.3	—	—
		D3-5	29.33	11.88	—	—
	*Halobacillus*	F1-26	52.25	4.2	—	—
		F1-13	41.18	66.2	15.92	27.24
	*Jeotgalibacillus*	D2-1	68.97	—	—	—
Proteobacteria	*Psychrobacter*	D1-1	27.6	32.65	14.98	2.18
	*Pseudoalteromonas*	F4-2	51.61	5.01	—	4.7
	*Shewanella*	H3-5	44.28	28.31	—	—

^a^Inhibition ratio refers to the difference between the relative residue activity of each sample after incubation with and without an inhibitor.

## Discussion

Investigating microbial diversity and functionality is of crucial importance in demonstrating “who's there and what do they do.” The culture-dependent method is the traditional way to explore and discover novel bacterial strains and products (Chen et al., 2003; Zhang et al., 2015). In recent years, the microbial diversity of environmental samples has been commonly investigated by culture-independent methods based on the rapid development of Illumina sequencing (Li Q. et al., 2022; Zhang et al., 2022). In this study, culture-dependent methods and culture-independent methods were interrelated for a better understanding of the bacterial diversity in the coastal mudflat, especially the protease-producing bacteria and their extracellular proteases.

The coastal mudflats are the transition zone of marine and terrestrial land, serving as an indispensable member of the global biogeochemical system. Excessive anthropogenic nitrogen input is a key factor influencing the coastal ecosystem (Li K. et al., 2019). Nitrogen loading caused eutrophication of the tidal flat environment, leading to hypoxia, red tides, and sediment loss (Cui et al., 2013; Cheng et al., 2020). In addition to inorganic nitrogen, environmental organic matters contain nitrogen mostly as protein. The organic feed and fertilizers applied in farming and aquaculture, animal feces and carcasses, as well as plant debris, contained a large amount of protein, an excess of which is an organic pollutant in coastal areas (Hodge et al., 2000; Davies et al., 2022). Soil enzymatic activity, especially soil hydrolase enzymes including protease, serves as a biological indicator to examine soil health (Maliang et al., 2020; Farooq et al., 2021). Microbes that inhabit coastal sediments play an important part in coastal biochemical cycling (Jiao et al., 2018). The protease-producing bacteria participated in the protection of coastal mudflats by secreting protease and degrading the protein in coastal mud.

It has been reported that nitrogen and phosphorus were released from an aquaculture farm and introduced pollution and eutrophication to the surrounding environments (Kawasaki et al., 2016). Consistently, in our study, the total concentrations of carbon, nitrogen, and phosphorus in the clam aquaculture area were 22.4 g/kg, 0.76 g/kg, and 0.62 g/kg, respectively, all of which were significantly higher than the corresponding values in the non-clam area of 9.64 g/kg (TC), 0.52 g/kg (TN), and 0.31 g/kg (TP). Recent studies have reported that 57% of nitrogen and 76% of phosphorus in aquatic feed were lost to the aquaculture water environment (Kong et al., 2020), and that the total nitrogen concentrations in the contaminated seawater of fishing harbors were two times higher than those of uncontaminated samples (Cheffi et al., 2022). All the related studies demonstrated that the fast-growing aquaculture industry had a great influence on the biogeochemistry factors of the surrounding areas.

We found that Proteobacteria was the most abundant phylum (44.47%) of the non-clam area in the coastal mudflat of Jiaozhou Bay, and the dominant class Gammaproteobacteria constituted 23.23% of the bacterial community. The dominance of Proteobacteria in the microbiota of various coastal environments has been reported in previous studies. Proteobacteria was found to be the most abundant phylum in two Atlantic coastal areas of France and Portugal, representing 89.3 and 82.3% of the total microbiota, respectively (Leite et al., 2017), as well as in the surrounding seawater of cultivation farms along coastal areas of the Yellow Sea (35.25%) (Ahmed et al., 2021), and in three Kerkennian fishing harbors (51.02–66.7%) (Cheffi et al., 2022). The bacterial community of Jiaozhou Bay sediments was also investigated, and the results showed that Proteobacteria was the most dominant phylum (61.3%), among which Gammaproteobacteria constituted the most abundant class (32.8%) (Liu et al., 2014). In addition, Proteobacteria and Gammaproteobacteria predominantly existed in the clam naturally growing area and the clam aquaculture area, with the composition of Proteobacteria accounting for 22.76 and 29.13%, as well as Gammaproteobacteria accounting for 16.37 and 17.51%. This was consistent with previous reports on the prevalence of Proteobacteria in the microbiota of marine invertebrates, such as oysters and hydroid (Fernández et al., 2013; Guo et al., 2017). On the other hand, we investigated the cultivable protease-producing bacteria in the studied areas and found an abundant bacterial community in the phylum Proteobacteria, especially in the class Gammaproteobacteria, which was affiliated with four genera *Pseudoalteromonas, Vibrio, Psychrobacter*, and *Shewanella*. *Proteobacteria* accounted for 41.18% in the non-clam area, 46.15% in the clam naturally growing area, and 27.78% in the clam aquaculture area of the total culturable protease-producing bacteria, which was consistent with the findings of the culture-independent approaches.

Members of Firmicutes were found as a minor phylum in the sediments of Jiaozhou Bay (Liu et al., 2014), which was consistent with our findings that Firmicutes only account for 0.37% of the microbial biomass in the non-clam area. However, the amount of Firmicutes increased to 1.85% in the clam naturally growing area and even to 21.03% in the clam aquaculture area. These findings are in line with the reports of Zhao et al. (2022), who observed Firmicutes as the most abundant phylum of the core bacterial communities in clams, with a composition of 26.2%. The prevalence of Firmicutes has also been shown in the microbiota of several marine invertebrates and aquatic environments of different geographic locations, such as the marine ark shell in the Japan Sea (Romanenko et al., 2009), the Easter oyster in the coastal Bay of USA (King et al., 2012), and the sponge in the Western Mediterranean Sea (Bauvais et al., 2015).

On the genus level, we found that *Woeseia, Loktanella, Trichococcus, Lutibacter*, and *Psychrobacter* were dominant genera in the studied mudflat areas. *Woeseia* genus was considered a core member of the microbial community in the marine ecosystem, which is characterized by the ability to assimilate inorganic carbon (Dyksma et al., 2016). *Woeseia* strains have been frequently isolated from coastal sediments and coastal seawaters (Du et al., 2016; Xu et al., 2022). *Loktanella* was reported to be a dominant genus in the coastal microbial community (Cardoso et al., 2019), and large numbers of *Loktanella* strains have been isolated from tidal flat sediments (Park et al., 2013, 2014; Tanaka et al., 2014). *Trichococcus* strains were characterized by their psychrotolerant ability, which was commonly isolated from environments at low temperatures such as cold spring (Zakharyuk et al., 2021). *Lutibacter* was reported to be predominant in shallow water marine sediment (Kerfahi et al., 2014), and some strains have been isolated from tidal flat sediments (Choi and Cho, 2006). However, except for a *Trichococcus pasteurii* strain that was isolated from freshwater crawfish and has been reported to produce alkaline proteases to degrade the myofibrillar protein (Qiu et al., 2022), no other studies report on the protease-producing ability of these four genera. What is different is the *Psychrobacter* genus. *Psychrobacter* strains were also known for psychrophilic characteristics, and some strains have been reported to produce cold-active protease (Amato and Christner, 2009; Perfumo et al., 2020). In this study, the *Psychrobacter* genus was found to be the third in abundance in sample H and the 35th in sample D based on the results of culture-independent methods. Correspondingly, as a result of culture-dependent methods, *Psychrobacter* was found to be a predominant genus of the analyzed culturable fraction in coastal mudflats, with an abundance of 17.65% in sample D, 5.6% in sample H, and 6.56% in total.

*Bacillus* genus, as a member of the phylum Firmicutes, has been frequently reported because of their bioremediation potentials of organic and inorganic compounds, which are also essential in the clam aquaculture area (Hsieh et al., 2020; Kumar et al., 2022; Liu et al., 2023). Zhang et al. (2015) isolated sixty-six protease-producing bacteria from Jiaozhou Bay sediments. Of all these cultivable protease-producing bacteria, *Bacillus* was found to be a major group (25.8%) (Zhang et al., 2015). Correspondingly, in our study, *Bacillus*-like bacterial communities in the class Bacilli and family Bacillaceae that were affiliated with five genera, including *Bacillus, Alkalihalobacillus, Rossellomorea, Halobacillus*, and *Cytobacillus*, accounted for 52.94% in the non-clam area, 30.77% in the clam naturally growing area, and 50% in the clam aquaculture area. Analogously, *Bacillus*-like species were also found as one of the dominant groups in other marine habits, e.g., sediments of mangrove wetlands in Hainan, China (Liu et al., 2017), the coast of South India (Sinimol et al., 2016), and the continental slope of eastern Arabian Sea (Farha et al., 2019). In addition, members of the genus *Bacillus* and related genera were widespread in terrestrial environments and exhibited rapid growth in high protein media (Manktelow et al., 2020), which may also cause the high composition of *Bacillus*-like species isolated from the studied areas, originating from surrounding terrestrial habits.

Additionally, according to the inhibitor assay on protease, we found that serine protease and metalloprotease were secreted by the isolated bacteria of the studied area as the principal types of proteases, generally similar to previous studies on sediments of the Antarctic Sea, sediments of the South China Sea, soils of the Antarctica South Shetland Islands, and sediments of Jiaozhou Bay (Zhou et al., 2009; Hou et al., 2013; Zhang et al., 2015; Liu et al., 2021). Relatively few strains isolated from Jiaozhou Bay sediments were found to secrete cysteine proteases (Zhang et al., 2015), and a minority of bacterial strains isolated from the soils of the Antarctica South Shetland Islands were reported to secrete aspartic and/or cysteine proteases (Liu et al., 2021). In this study, we also found that a small quantity of aspartic protease and cysteine protease was produced by a few strains isolated from the non-clam area and the clam naturally growing area. These bacterial extracellular protease findings may shed light on the understanding of organic nitrogen degradation in coastal mudflat areas and the development of microbial protease agents to prevent pollution and eutrophication.

## Conclusion

In summary, this study analyzed the bacterial taxonomy composition of different clam-growing areas in the coastal mudflats of Jiaozhou Bay, especially protease-producing bacteria and their extracellular protease. The results showed that the diversity of the bacterial community and the protease-producing bacteria of the clam naturally growing area was higher than that of the non-clam area and the clam aquaculture area. *Bacillus*-like species, including the genera *Bacillus, Alkalihalobacillus, Rossellomorea, Halobacillus*, and *Cytobacillus*, were the dominant cultivated protease-producing groups in the Jiaozhou Bay coastal mudflats, and serine proteases and metalloproteases were the principal types of proteases produced by the bacteria. These findings contribute to a better understanding of the function of protease-producing bacteria in organic nitrogen degradation in coastal mudflat areas and the development of bacterial protease agents to improve the coastal aquaculture quality.

## Data availability statement

The datasets presented in this study can be found in online repositories. The names of the repository/repositories and accession number(s) can be found in the article/Supplementary material.

## Author contributions

JY designed the research and wrote the manuscript. ZL and XG conducted the research. GL collected samples, carried out bioinformatic analyzes, and revised the manuscript. YL, NJ, XX, and QS contributed ideas to the study. All authors contributed to the article and approved the submitted version.
