# Supplementary material for: Diversity of the protease-producing bacteria and their extracellular protease in the coastal mudflat of Jiaozhou Bay, China: in response to clam naturally growing and aquaculture
Source: Front Microbiol. 2023 May 19;14:1164937. doi: 10.3389/fmicb.2023.1164937 (PMC10236810; doi:10.3389/fmicb.2023.1164937)
Supplement: Supplementary file 1 [file Data_Sheet_1.docx]

**Supplementary Material**

**Title:** Diversity of the Protease-producing Bacteria and their Extracellular Protease in the Coastal Mudflat of Jiaozhou Bay, China: in Response to Clam Naturally Growing and Aquaculture

**Running Title：**Microbial Diversity in Coastal Mudflat

Zhiyun Liu^1^, Guangchao Liu^2^, Xuzhen Guo^1^, Yang Li^1,3^, Na Ji^1,3^, Xingfeng Xu^1,3^, Qingjie Sun^1,3^, Jie Yang^1,3*^

**Affiliations**

^1^College of Food Science and Engineering, Qingdao Agricultural University, Qingdao 266109, People's Republic of China;

^2^College of Life Science, Qingdao Agricultural University, Qingdao 266109, People's Republic of China;

^3^Qingdao Special Food Research Institute, Qingdao 266109, People's Republic of China.

*CORRESPONDENCE
Jie Yang
[phdyang1314@163.com](mailto:phdyang1314@163.com)


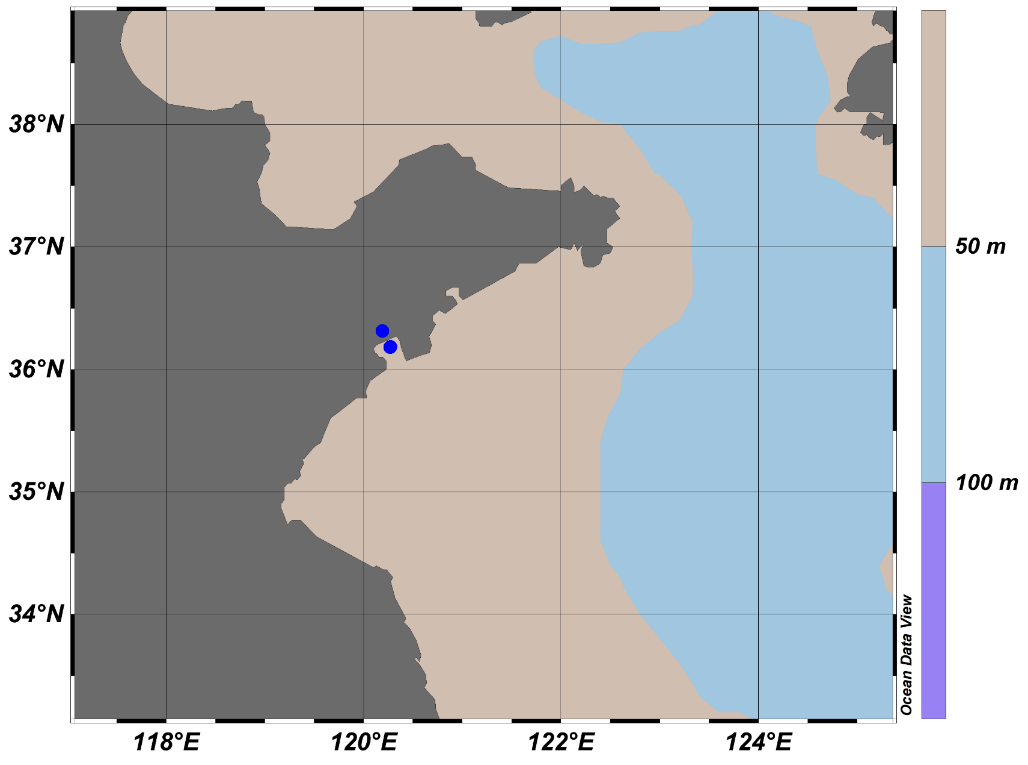


**Supplementary Figure S1.** Geographic location of sampled stations in coastal mudflat of Jiaozhou Bay


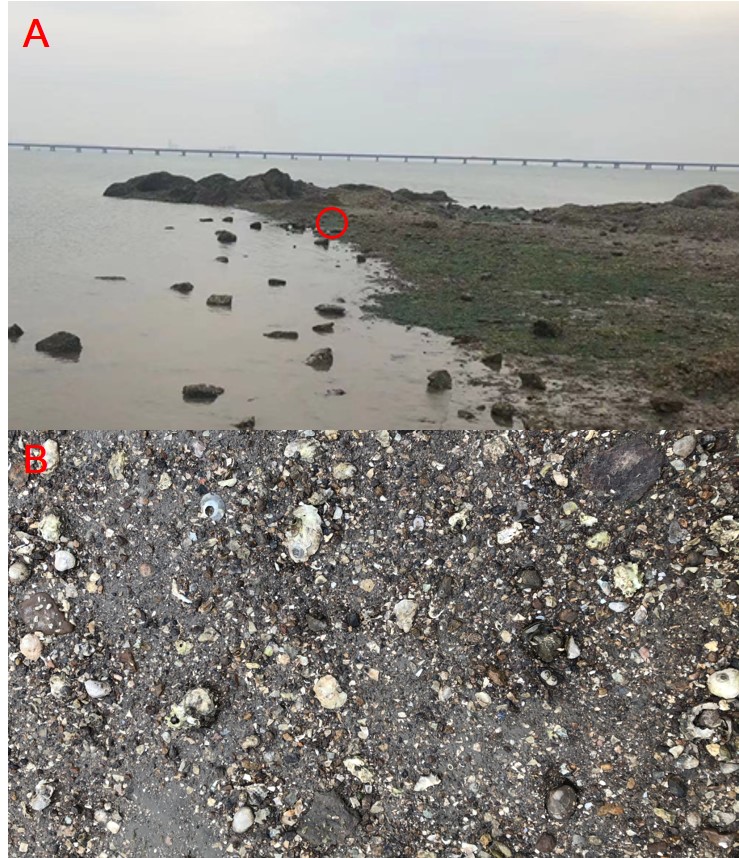


**Supplementary Figure S2.** Sampling site of non-clam area (A) and in situ mud during the low tide period (B).


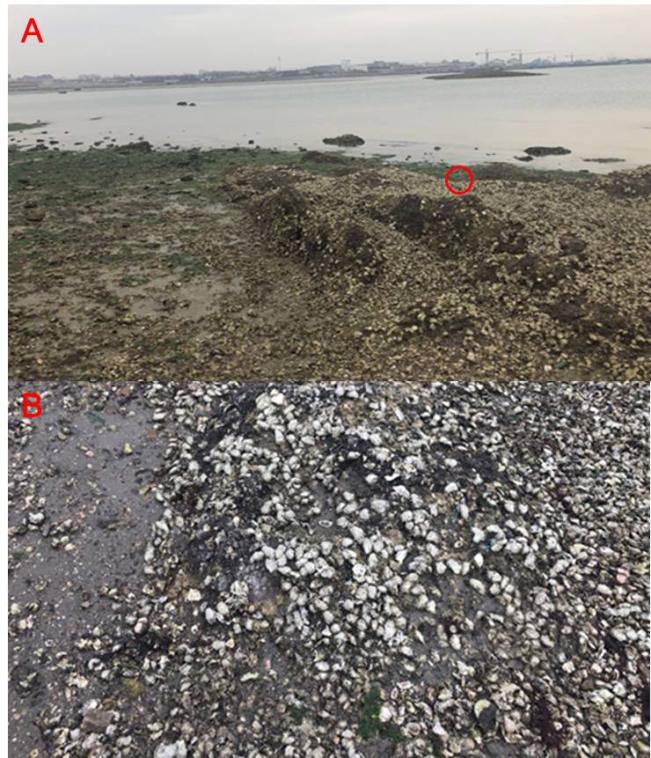


**Supplementary Figure S3.** Sampling site of clam naturally growing area (A) and in situ mud during the low tide period (B).


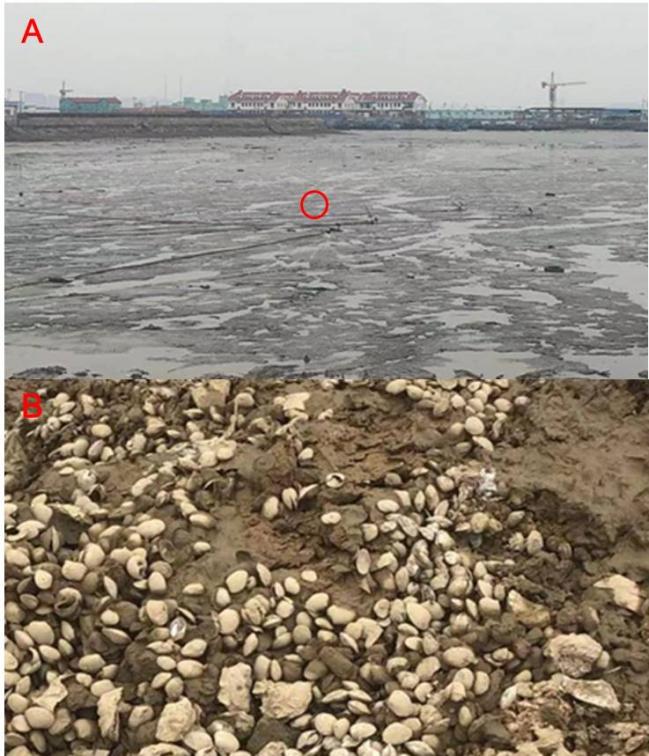


**Supplementary Figure S4.** Sampling site of clam aquaculture area (A) and in situ mud during the low tide period (B).


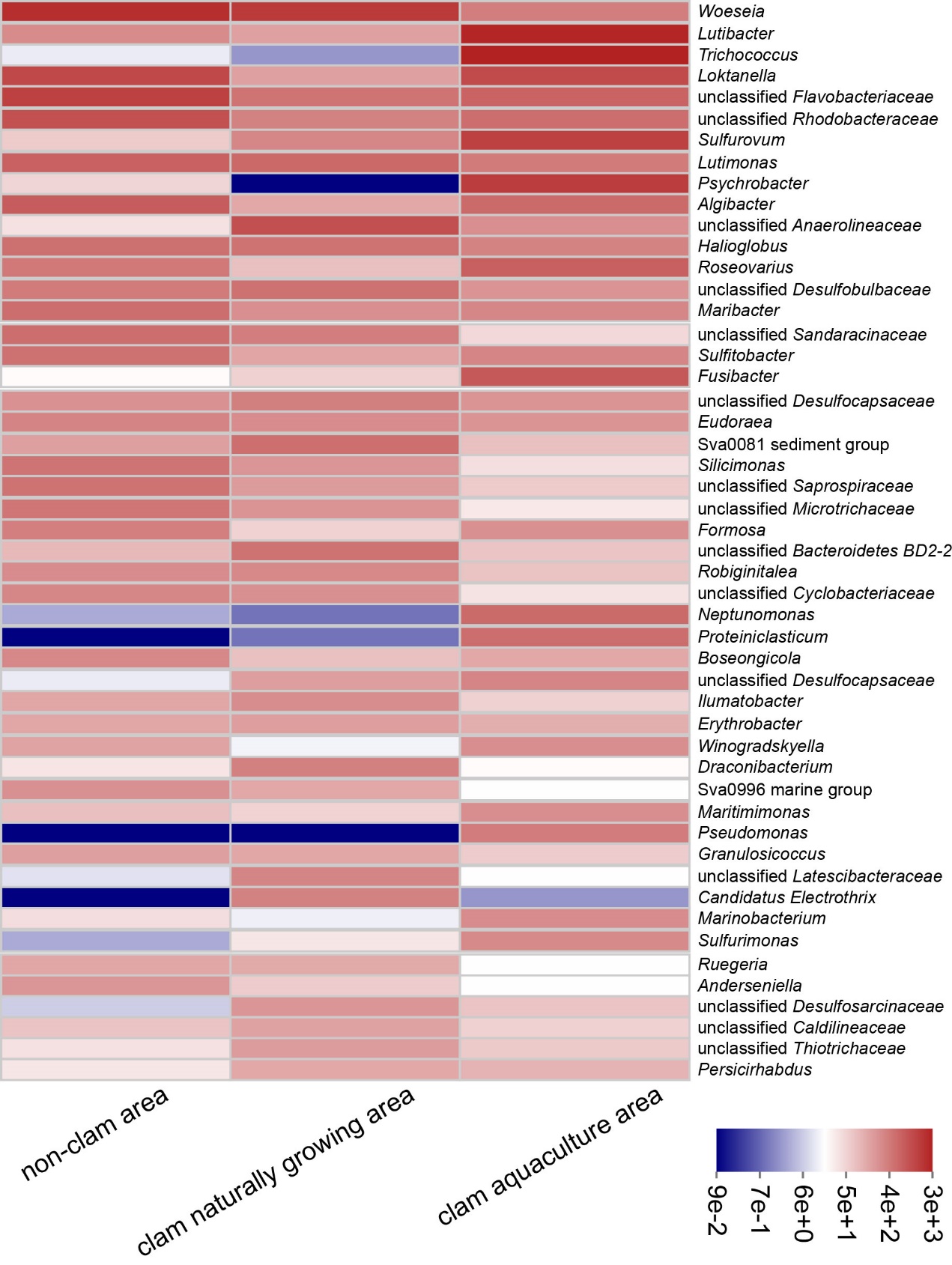


**Supplementary Figure S5.** Community heatmap analysis of the mud samples on Genus level. The mud samples were taken from non-clam area (sample D), clam naturally growing area (sample F) and clam aquaculture area (sample H), and the microbial community was investigated by culture-independent methods. The heatmap was generated from the top 50 abundant OTUs according to relative abundance which was represented in different colors.

**Supplementary Table S1.** GenBank accession No. of strains

| **Bacterial strains** | **GenBank accession No.** | **Bacterial strains** | **GenBank accession No.** |
| --- | --- | --- | --- |
| D1-3 | OQ625903 | H2-13 | OQ626210 |
| D1-12 | OQ625916 | H3-29 | OQ626214 |
| D1-31 | OQ625941 | D2-6 | OQ625907 |
| D1-23 | OQ625938 | F4-1 | OQ625994 |
| F1-12 | OQ626004 | F3-13 | OQ626013 |
| F4-9 | OQ625995 | F3-4 | OQ626012 |
| H1-7 | OQ690495 | F4-10 | OQ625996 |
| H2-21 | OQ642131 | F3-3 | OQ626011 |
| H2-15 | OQ626228 | F3-24 | OQ625993 |
| H2-22 | OQ626221 | F3-2 | OQ626010 |
| H2-9 | OQ629994 | F4-2 | OQ626014 |
| H2-6 | OQ626209 | F4-8 | OQ626017 |
| H2-10 | OQ629902 | H3-3 | OQ626212 |
| H2-14 | OQ629890 | D2-13 | OQ625909 |
| D1-8 | OQ651245 | D3-8 | OQ625912 |
| D2-9 | OQ625908 | D1-1 | OQ625902 |
| D1-9 | OQ625921 | H1-5 | OQ626207 |
| F1-2 | OQ626000 | D3-7 | OQ625911 |
| F2-3 | OQ626001 | D3-5 | OQ625910 |
| H2-1 | OQ626208 | F1-6 | OQ626002 |
| F1-8 | OQ626003 | H3-5 | OQ626213 |
| F2-6 | OQ626008 | H3-30 | OQ626220 |
| F2-12 | OQ629993 | H3-1 | OQ626211 |
| F2-5 | OQ629992 | D2-4 | OQ625939 |
| F1-13 | OQ626005 | D2-2 | OQ625906 |
| F1-26 | OQ626006 | D2-3 | OQ625917 |
| D2-1 | OQ625905 | F4-3 | OQ626015 |
| F4-5 | OQ626016 | F4-11 | OQ626018 |
| F1-30 | OQ626007 | F4-16 | OQ626019 |
| H1-3 | OQ626206 | F2-9 | OQ626009 |
| H1-2 | OQ626205 |  |  |

**Supplementary Table S2.** H/C ratio of the protease-producing strains on protein substrate plates.

| **Genera** | **Strains** | **H/C ratio** | | |
| --- | --- | --- | --- | --- |
|  |  | **Milk Powder**  (1.0%) | **Casein**  (0.5%) | **Gelatin**  (0.5%) |
| *Alkalihalobacillus* | D1-3 | 3.04 | 3.07 | 2.2 |
|  | D1-12 | 3.45 | 5.17 | 2.9 |
|  | D1-31 | 2.74 | 2 | 1.52 |
|  | D1-23 | 3.46 | 2.54 | 1.67 |
|  | F1-12 | 3.44 | 2.75 | 2.65 |
|  | F4-9 | 5.37 | 4.21 | 4.68 |
|  | H1-7 | 5.21 | 4.45 | 4.79 |
|  | H2-21 | 2.5 | 2.09 | 1.86 |
|  | H2-15 | 3.45 | 2.3 | 1.9 |
|  | H2-22 | 3.89 | 2.78 | 1.94 |
|  | H2-9 | 4.8 | 3.47 | 2.8 |
|  | H2-6 | 3.83 | 2.72 | 2.17 |
|  | H2-10 | 3.48 | 2.7 | 2.09 |
|  | H2-14 | 3.59 | 2.91 | 2.18 |
| *Bacillus* | D1-8 | 7.89 | 9.5 | 3.29 |
|  | D2-9 | 2.94 | 1.24 | 5.13 |
|  | D1-9 | 3.7 | 3.5 | 2.92 |
|  | F1-2 | 7 | 7.57 | 3.53 |
|  | F2-3 | 5.43 | 3.33 | 2.95 |
|  | H2-1 | 4.09 | 3.27 | 2.45 |
| *Cytobacillus* | F1-8 | 4.08 | — | 3 |
| *Dokdonia* | F2-6 | 3.22 | 1.86 | 3 |
| *Flavihalobacter* | F2-12 | 2.67 | 2.88 | 3.38 |
| *Gramella* | F2-5 | 2.5 | 1.83 | — |
| *Halobacillus* | F1-13 | 5.07 | 4.17 | 5.13 |
|  | F1-26 | 1.67 | — | 1.93 |
| *Jeotgalibacillus* | D2-1 | 5.77 | 4.54 | 1.56 |
| *Lacticaseibacillus* | F4-5 | 2 | 1.95 | 1.7 |
| *Planococcus* | F1-30 | 1.81 | — | 2.08 |
|  | H1-3 | 4.33 | 5.56 | 3.17 |
|  | H1-2 | 3.18 | 2.02 | — |
| *Metaplanococcus* | H2-13 | 3.11 | 2.14 | — |
| *Pontibacter* | H3-29 | 1.8 | 1.9 | 1.45 |
| *Pseudoalteromonas* | D2-6 | 2.28 | 1.89 | 1.17 |
|  | F4-1 | 2.94 | 1.94 | 1.28 |
|  | F3-13 | 2.5 | 1.64 | 1.95 |
|  | F3-4 | 2.17 | 1.33 | 2.21 |
|  | F4-10 | 1.77 | 1.77 | 1.82 |
|  | F3-3 | 2.13 | 1.74 | 1.87 |
|  | F3-24 | 1.67 | 1.76 | 2 |
|  | F3-2 | 2.45 | 2.4 | 1.5 |
|  | F4-2 | 2.05 | 2.16 | 1.68 |
|  | F4-8 | 2.18 | 1.82 | 1.68 |
|  | H3-3 | 2.86 | 2.72 | 1.95 |
| *Psychrobacter* | D2-13 | — | — | 7.75 |
|  | D3-8 | — | 1.89 | — |
|  | D1-1 | 3.47 | 4.39 | 5.67 |
|  | H1-5 | 7.43 | — | 6.67 |
| *Rossellomorea* | D3-7 | 4 | 3.36 | — |
|  | D3-5 | 1.9 | 1.82 | — |
|  | F1-6 | 3.75 | — | 1.86 |
| *Shewanella* | H3-5 | 2.58 | 2.58 | 2.05 |
|  | H3-30 | 2.75 | 2.95 | 1.9 |
|  | H3-1 | 3.15 | 3.25 | 2.35 |
| *Vibrio* | D2-4 | 4.47 | 3.11 | 2.05 |
|  | D2-2 | 3.82 | 3.09 | 2.05 |
|  | D2-3 | 3.81 | 3.33 | 2.24 |
|  | F4-3 | 3.45 | 3 | 1.6 |
|  | F4-11 | 4 | 3.45 | 1.95 |
|  | F4-16 | 2.46 | 2.46 | 1.67 |
| *Zobellia* | F2-9 | 1.84 | 2.38 | 1.55 |
